# Supplementary figures and images for: Estimating plant biomass in agroecosystems using a drop-plate meter
Source: PeerJ. 2023 Aug 2;11:e15740. doi: 10.7717/peerj.15740 (PMC10404029; doi:10.7717/peerj.15740)

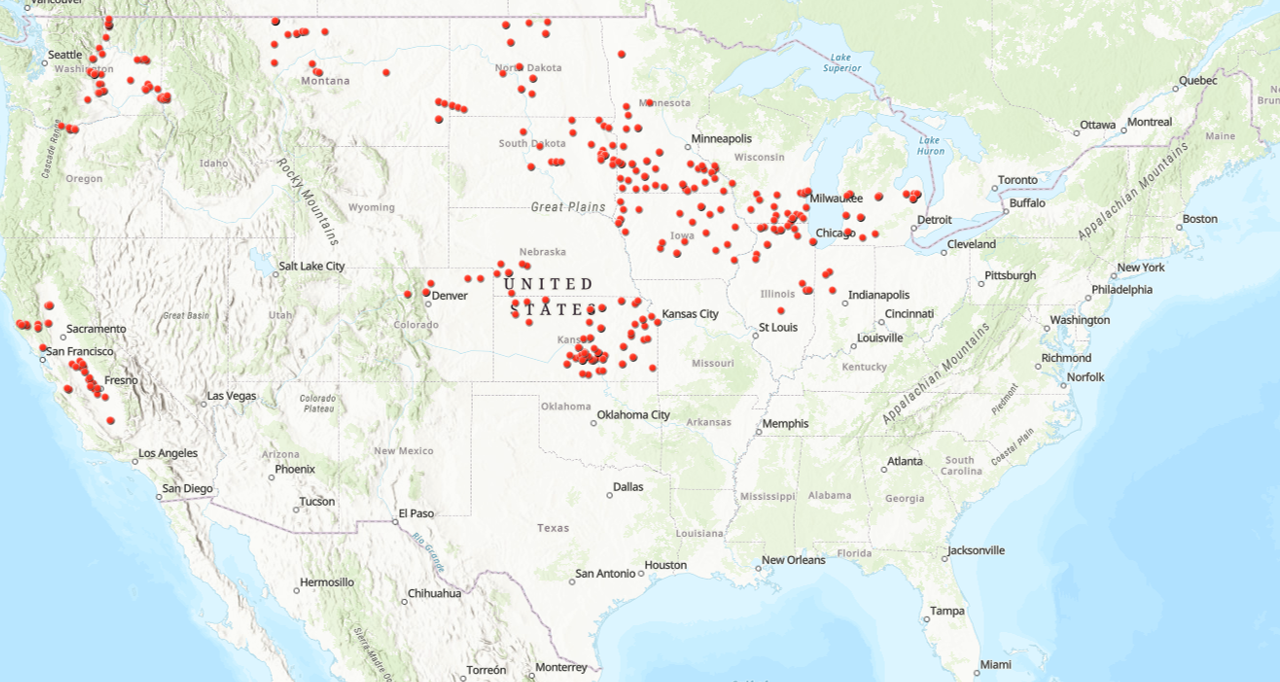

Supplement: Supplemental Information 2 [file peerj-11-15740-s002.png]
